# Embracing the cordillera: Luis Carranza Ayarza and the development of environmental imaginaries in late-nineteenth-century Peru

**DOI:** 10.1590/S0104-59702023000100006en

**Published:** 2023-04-03

**Authors:** José Ignacio Mogrovejo Palomo

**Affiliations:** i Teaching assistant, Pontificia Universidad Católica del Perú. Lima – Perú jimogrovejo@pucp.edu.pe

**Keywords:** Luis Carranza Ayarza (1843-1898), Geographical Society of Lima, nature/culture divide, environmental imaginary, modern Peru, Luis Carranza Ayarza (1843-1898), Sociedad Geográfica de Lima, división naturaleza/cultura, imaginario ambiental, Perú moderno

## Abstract

This article examines the relationship between local scientific ideas about the natural world and the economic potential to transform the modern nation-state in Peru during the late nineteenth century. Writings by the Peruvian scientist Luis Carranza indicate how support for a distinctive environmental imaginary of the country’s geography made it possible to conceptualize nature as an essential component of Peruvian identity. As a result, local scientists had to “imaginatively” reshape the nature of the Andes for modernization purposes. The social and political ramifications of these ideas in Carranza’s work were key to the foundation of scientific institutions such as the Geographical Society of Lima.

## Resumen

Este artículo examina la relación entre las ideas científicas locales sobre el mundo natural y el potencial económico para transformar el Estado-nación moderno en el Perú a fines del siglo XIX. Los escritos del científico peruano Luis Carranza señalan cómo el apoyo a un imaginario ambiental distintivo de la geografía del país permitió conceptualizar la naturaleza como componente esencial de la identidad peruana. Como resultado, los científicos locales tuvieron que remodelar “imaginativamente” la naturaleza de los Andes con fines de modernización. Las ramificaciones sociales y políticas de estas ideas en la obra de Carranza fueron clave para la fundación de instituciones científicas como la Sociedad Geográfica de Lima.

One day in mid-1898, the Royal Geographical Society headquarters in London received word that the Peruvian physician and geographer Luis Carranza Ayarza had died. This news would have greatly upset Sir Clements Markham, the most *Peruanista* member of the institution and a personal friend of the deceased. Likewise, news of Carranza’s death would also have been disconcerting to Peru’s political and scientific elites of that time; they honored his memory as a proactive force in the parliamentary arena and as the founder of the Geographical Society of Lima, the country’s first modern scientific institution. These tributes clearly indicated that Carranza’s death left a significant void in local knowledge communities among those who knew him, and would also be a fundamental moment in the national institutionalization of modern science (C.J.B, 1898; The Royal…, 1898).

Despite Carranza’s contribution to modernization efforts in Peru, little attention has been paid to his scientific work, which reflected a wide array of interests including meteorology, medicine, geography, anthropology, and economics, to name just a few (Caravedo, 1941; Gamarra, 2014; Gootenberg, 1993; Núñez, 1988; Thurner, 1997). The lack of studies on Carrazza’s life and work leaves a gap in the historiography of modern scientific practices in Peru during the late nineteenth century. His work also serves as an example of what historians Marcos Cueto and Leoncio López-Ocón have identified as a distinctive mark of modern Peruvian science: the territorial ideology of geographical nationalism. Embodied in the Geographical Society of Lima, this local scientific perspective not only made it possible to recognize the country’s internal and external frontiers, but it also aligned different disciplines in the study of natural resources to commodify the unexploited wealth of the Andean territories for economic purposes (Cueto, 1992, p.35-41; López-Ocón, 2001, p.1-8). In addition to supporting capitalist endeavors, this vision paved the way for a specific conceptualization of Peru’s entire geography, including elements ranging from the mountains to the Indigenous populations. The sociocultural manner in which these elements were conceptualized made them legible to the postcolonial modern creole imagination. In this sense, the concept of *cordillera* (“mountain range”), a term commonly found in intellectual production during the Republican period, acted as a synecdoche for the distinctive geography of the Peruvian highlands. This distinctive geography was racialized by the coastal *Limeño* elites who imposed a specific hegemonic order on the country’s social fabric (Méndez, 2011; Orlove, 1993).

Although the intellectual history of “Nature” has produced a vast bibliography focusing on the creation of local knowledge about the natural world, recent works about the conception of “the environment” as an idea situate its genesis in debates involving naturalists, sociologists, biologists, and environmentalists in different eras and at different scales of observation. This intellectual tradition has allowed us to imagine planet-wide dangers to humans and nonhumans alike from climate change, for example. But in trying to explain the origins of contemporary concerns about the consequences of climate change, we have reinforced the predominance of ideas from the Global North in the historicization of the natural world between the eighteenth and twenty-first centuries (Benson 2020; Warde et al., 2019). This further problematizes what Dipesh Chakrabarty has recognized as the limits of the nature/culture dualism proposed by Bruno Latour. This divide describes how the processes of modernity and capitalism filter “nature” into diverse anthropocentric units, although Chakrabarty (2021, p.106) emphasizes how the discussion about the nature/culture division from the West “found a fresh and original articulation in the imagination of the colonized.” Even though his reasoning addresses anticolonial modernization in India, the critique can also be creatively applied to postcolonial Latin America. The newly formed nations in this region attempted to consolidate their autonomy and the ideal of social equity by incorporating scientific practices into their bureaucratic and productive realms. In their quest to commodify the natural world, they used flexible modes of producing knowledge, and as modern science was institutionalized in Peru specific environmental imaginary helped local scientific communities achieve their goals of political legitimacy in the eyes of the state (Saldaña, 2006; Scott, 1998, chap.1).

In this essay, I use the term “environmental imaginary” to recognize how nature is represented by the diverse forms of social relationships and uses of knowledge within a community to guide interventionist approaches toward the natural world (Watts, Peet, 2004). This is one way to standardize the social production of nature to legitimatize the formation of communities of expertise, which were able to imbue their representations with specific cultural values. Drawing on the geographer Derek Gregory (2001, p.88-96), it is part of a process that made the modern framing of “nature” possible, allowing it to be excised from and represented as external to the historical trajectories of “culture.” This makes it possible to commodify the natural world and allows the potential implementation of systems of regulation, surveillance, and enforcement.

From Latin American historiography, works about colonial or postcolonial science have highlighted how interactions with the landscapes and biota molded the imperial experiences of European powers and state interventions in the natural world. Nevertheless, as historian Mark Carey (2009, p.229-230) contends, there has not been much interest in problematizing the “cultural” aspect of material environments. In the science and technology studies literature about the region, “creole science” has been characterized as an easy-to-use term that is too broad in how it casts *criollo* knowledge production about the natural world. It does, however, indicate the common understanding among creole scientists of the time about how external factors influenced local processes of domesticating nature (Cañizares-Esguerra, 2002; Cushman, 2011; McCook, 2002). Studies related to the historical trajectories of “nature” in Latin American societies have noted the different circumstances in which political elites used knowledge of the natural world to hegemonize ecological and social differentiation. In the case of Colombia, for example, Nancy Applebaum (2016) analyzed how the Chorographic Commission of New Granada conducted fieldwork in search of ideal “homogeneous” populations and landscapes but found fragmented territories and diverse peoples. The heterogeneity of these territories and peoples was placed at the disposal of Bogotá-based elites to consolidate their political and economic hegemony in the mid-nineteenth century. In the case of Mexico, Christopher Boyer (2015) discovered how forests were spaces of uneven negotiation, revealing a process of politicization between technocratic elites and local Indigenous people. This suggests that because of the contested coexistence between “superior” scientific and “native” knowledge, forests became landscapes capable of molding national ecological consciousness in the twentieth century. In the case of Brazil, a country with ecological diversity of continental proportions, José Pádua (2018) has argued that political and intellectual elites envisioned an economic-driven territorial occupation of places like the *sertão* backlands, which the elites believed to be barely occupied by “civilization.”

Building on these perspectives, I argue that the development of a specific environmental imaginary of the Andes portraying its population and the natural world as flawed but potentially redeemable and purposeful was fundamental in shaping the ideal of development envisioned by Peru’s *civilista* elite in the late nineteenth century. The elite class used discursive appraisals in the scientific press to domesticate Andean landscapes and defend the country’s climates against Eurocentric beliefs of a “pathologized” geography. Molded to correspond to the *civilista* elite’s ideals, these particular tropes regarding nature also envisioned how the latent capabilities of the Indigenous people from the highlands could be incorporated into the modernizing aspirations of political and scientific elites. As a representative of this ruling class, Luis Carranza sought to normalize a modern condition of “nature” in his writings. The Andean environment was an object that needed to be dominated, occupied, and exploited to consolidate the Peruvian nation-state, an idea that would be perpetuated in the work conducted by the scientific institution Carranza left as his legacy.

## A tool towards progress: Peruvian science in the mid-nineteenth century and its context

After the War of Independence, parliamentary and intellectual figures sought to make a space for themselves in a region that was both economically and politically devasted after Spanish rule. One of the main pillars of economic liberalism was to develop solid foundations for scientific practices, which involved the participation of scientists like Mariano de Rivero to promote state-backed mining enterprises. At the same time, there was pressure from foreign sources like English companies eager to invest in the economies that were emerging at the time. These financiers enticed local businessmen and parliamentary figures with the promise of capital flow and new technologies to recuperate the extractive activities that were key during colonial times but also seen as central to the economies of the newly-liberated regimes.^1^ This complex situation was part of a larger scheme in the beginnings of postcolonial life for Latin American nations as British capitalists sought to create an informal empire of economic dependence in the nascent Andean republics. For this reason, support for scientific activities occurred haphazardly, manifesting only as isolated efforts or short-lived initiatives rather than a systematic, collective, and society-wide approach (Deustua, 2017, p.63-68; Seiner, 2004, p.111-118).

Nevertheless, some degree of political stability allowed for the improvement of pre-existing scientific institutions. In the medical profession, after the end of medical reforms of the Bourbon era and the beginnings of the Republican period, the weighty colonial legacy of the Tribunal del Protomedicato (the most important entity supervising medical practice in the Spanish American Empire) represented an obstacle to the anticolonial and liberal scientific elites who yearned for modernization. However, their limited success in reforming the country’s foremost medical institution wasn’t because they were impervious to criticism against the curricula as outdated and the lack of scientific innovation on the part of Lima’s physicians. It was because they couldn’t detach themselves from the nationalist authoritarianism that had permeated the administration and teaching of medical practices, which rendered anyone without a license from the Protomedicato an illegal healer. Being deeply rooted in contradictory affairs, this medical-political institution couldn’t fulfill the modernizing aspirations of Lima’s creole physicians and was eventually disbanded Unfortunately, this medical-political institution was unable to fulfill the modernist aspirations of Lima’s creole physicians and was eventually disbanded (Jouvé Martin, 2014, p.94-95, 120-124; Warren, 2010, p.211-217).

Despite an initial setback, winds of optimistic change arrived between the 1840s and 1860s during the Guano Era, when public officials had sufficient financial means to implement several changes in political and economic administration. This created the liberal basis of a modern bureaucratic system, and along with it, a major urban refashion and remodeling. Besides satisfying the material ideals of the elite, resources were spent to create broad support for the scientism embodied in public engineering works. As part of the improvements made to the educational system, Lima’s decaying School of Medicine was re-opened through the efforts of the physician Cayetano Heredia, who pushed to reform it in order to create an institution capable of centralizing the public health duties of the state as well as to monopolize the administration of medical activity (Gootenberg, 1993, p.58-63; Lossio, 2002, p.56-57). These improvements shed a positive light upon the fact that by this time, Lima’s medical union now had an official gazette to intervene in public debates about Peruvian society, believing it was beneficial for the advancement of “national science.” This marked an important departure from their previous attitude of restricting their discussions and lectures to only a direct academic audience (Sociedad…, 1856).

To be sure, the compromise made to permit more public use of scientific practices was not solely focused on medicine; although control over sanitary policies was a main concern for the scientific elite, there was also a broader need to train citizens in natural sciences and engineering. The foundation of the Science Faculty of Lima in 1866 was intended to meet this need by training students for medical careers or as technical professionals, even if this latter function was only achieved after the creation of the School of Engineers in 1876. Nevertheless, there was wide support for the promise of science as a “tool towards progress” because it helped landowners accumulate capital and thereby increase their political and social precedence over Indigenous territories. This contributed to further modernization of the liberal state by bureaucratizing traditional sectors and reassuring that liberty would prevail in altering the social order and eventually replacing it with a bureaucratic and homogenous culture (Cueto, 1989, p.66; Weinberg, 1998, p.52-55). These ideas are expressed in the opening address given by the president of San Marcos University, Juan Antonio Ribeyro, in 1870, in which he emphasized the need to reinvigorate this long-standing institution with a shift to focus more on the natural sciences than literary arts. Ribeyro (1871, p.21-24) was also an ideologue of the Partido Civil and understood that mining, agriculture, and civil engineering were representative of “superior” scientific knowledge and should be prioritized as the means to maintain the freedom of societies. This same optimism led our protagonist Carranza to travel to Peru’s capital and pursue higher education.

Born in Ayacucho in 1843, Luis Carranza Ayarza was the son of a military officer and went to live with an aunt in Lima. This aunt was married to Manuel José de Amunátegui y Muñoz, the founder of one of the most important newspapers of the Guano Era, *El Comercio*. This later had an enormous impact on Carranza’s life: from 1875 to his death, Carranza served as the newspaper’s editor-in-chief. During his career as a journalist, this newspaper served as one of the main channels of expression for the Peruvian intellectual elite (Caravedo, 1941, p.11). Once in the capital, he enrolled in the most prestigious and modern educational institution in Peru, the Colegio de Guadalupe, which according to Caravedo (1941, p.8-9) was the place where Carranza’s quest for knowledge took form, something that likely had to do with the new liberal focus on education in the country.

After finishing his basic education, Carranza was determined to pursue professional studies in physiology. He attended Lima’s School of Medicine and graduated in 1866 with a bachelor’s degree. In his thesis, he supported Louis Pasteur’s novel theory of germ contagion in his analysis of the causes of infectious diseases through the action of fermentation in diverse environmental settings (Carranza, 1866). His awareness of Pasteur was probably the result of the common perception among physicians in Latin America at the time venerating French medicine as the vanguard of physiological knowledge. Carranza’s level of determination regarding medical theory was probably unusual for a young graduate. At the time, there were polarized debates in the medical community about sources of contagion, which had far-reaching implications on the design of public health policies to control contagious diseases like yellow fever. While many physicians promoted the use of quarantines and lazarettos to contain person-to-person contagion, others encouraged the eradication of the environmental foci of diseases. For his part, Carranza supported the idea that diseases were caused by microscopic particles in the air, aligning himself with the biomedical vanguard. This was a controversial stance since many of the older and more experienced members of the medical community in Peru had a traditional view of etiology (Cueto, 2003, p.321-322; Lossio, 2002, p.58-68).

Carranza was self-trained in several disciplines and had a broad, holistic view of the natural world that surpassed the pragmatic goals of his immediate community of knowledge. According to Núñez (1988, p.8-10), this had much to do with the buoyant nature of local scientific culture, primarily represented by the medical profession and naturalist studies concerned with finding practical solutions to the many societal issues that impacted Peru. This was a singular part of larger efforts to comprehend the role of humans in their domestic environment and in the social groups to which they belonged; still, it involved what was fundamentally an overly broad view of the social reality in Peru, and a more nuanced comprehension was required. Finding this level of understanding of Peru’s problems involved broadening the basis of scientific rationale to include an element of patriotic pride that might serve as an ontological crossroads for the study of the country’s issues. Fortunately for Carranza, the works of one of the most important *Peruanista* naturalists provided an outlet to address such concerns during the second half of the nineteenth century.

## The “Raimondian” blueprint

In 1862, a tall, skinny, languid-looking man from Milan named Antonio Raimondi walked among a crowd of young *Sanmarquinos* who were excited to see this same Raimondi give the opening lecture of the academic year. In it, the Italian evoked the memory of past naturalists and local scientist-explorers to captivate his audience, urging them to uncover the hidden richness of the natural world as the main gateway to Peru’s prosperous future. Raimondi (1862, p.196-224) also lauded the local erudite community and encouraged his audience to recognize the importance of their past achievements for the broader understanding of nature. Unsurprisingly, Raimondi’s ideas resonated beyond the classrooms of San Marcos and significant enthusiasm among colleagues who founded the Geographical Society of Lima, for example. His ideas were also influential among the *civilista* politicians of the 1870s who financed the publication of Raimondi’s magnum opus, *El Perú*. At his funeral in 1891, Carranza (1891, p.29-30) stated that Raimondi’s legacy as the “first and greatest of all sages” would cultivate an emotional bond between knowledge and the *Patria*. Looking at his work, young graduates had a life experience to follow in order to insert themselves in the annals of national science. In that sense, the “Raimondian blueprint” would serve as an inspiration for future generations of scholars to study the natural world.

By incorporating the Italian naturalist into the collective memory of present and future Peruvian communities of expertise, Carranza also outlined an ideal model to replicate and produce the “geographical consciousness” of the national territory (Miller, 2020, p.147-148). When the first part of *El Perú* was published in 1874, political and economic elites had adopted a specific agenda emphasizing the interrelationship between science and governability in political and social areas. In the times of the “República práctica,”^2^ finding other ways to produce revenue and not depend solely on guano was a primary objective for the “Partido Civil,” which wanted to ensure the continued viability of its political project. Perceived as a critical moment for the national economy, the downfall of the export model led to greater public intervention in financial markets and desperate measures to promote saltpeter extraction. Nevertheless, the constitution of a commercial and financial bourgeoisie in Lima narrowed the interests of the elites by focusing on developing a strong state, which may have left the impression that such policies promoted decentralization of capital by calling for the construction of roads and railroads through the hinterlands and instituting that reinforced local rights over domestic politics. But when a matter was in fact of “national transcendence,” there were few limits on central state intervention in regional issues (McEvoy, 2017, chap.3; Mücke, 2010, p.65-68).

After firmly establishing a remarkable reputation as an explorer and professor of natural history at San Marcos University, Raimondi was responsible for discovering the location of guano and saltpeter deposits in the 1850s. He also wrote elaborate encyclopedic monographs detailing the main characteristics of the different regions of Peru. The most important of them, *El Perú*, provided leaders with practical knowledge they could use to better exploit their natural resources (Seiner, 2003, p.520-522; Villacorta, 2008, p.235-240). As explained by Matthew Himmley (2020, p.235-240), Raimondi’s emphasis on the “richness” contained within the mineral deposits in the hills of Ancash served as a historical revindication of mining as a source of economic revitalization that would be complemented by the anticipated completion of a northern railroad to scale up local production. These practical motivations were fundamental to the main principles of Raimondi’s scientific thought. Both supporters and critics promoted the synthesis of his main principles and contributions to Peruvian science, and Carranza was no exception.

In a book review published in 1875, the first of two aspects Carranza highlighted from Raimondi’s work was the detailed observations and measurements made by the Italian naturalist despite the adverse circumstances, and also how this eventually granted him the privilege of achieving scientific excellency on local lands. By traveling across unsurmountable landscapes, the solitary work of Raimondi had the great merit of not only overcoming technological and practical difficulties in Peru’s rugged topography but also successfully making a scientific discovery in the steep highlands, a place that represented “the greatest source of knowledge.” Raimondi discovered a long- and multi-flowered plant in the immediacies of Huaraz and baptized it *Porruteia gigantea* (Carranza, 1887, p.72-75). For Carranza, the gigantic “object” that Raimondi heroically found embodied his scientific experiences in the Peruvian Andes, which had value not only as a novelty in itself but also as an “inscription” that was both mobile and immutable. Not only was the flowering plant available for introduction into local scientific circles as something “absent” and spatially distant from the capital, but it also embodied a dual “non-presence:” the flora and the *cordillera* (Latour, 1990, p.25-27). This fact demonstrated that it was possible to strike a path towards incorporating these far-off territories into the nation by embracing their “imagined” persona. If the virtues of these geographical hinterlands were to be considered, this also meant accepting the difficulties involved. This was to become the most distinguishable characteristic of Peru’s “natural” regions in the nineteenth-century imagination: their geographical accessibility.

Roads and communication networks were fragmented and scattered among coastal urban centers and the cities and towns that served as administrative centers in the highlands. Moreover, state efforts to develop a vast infrastructure of railroads to interconnect ports and cities with mines and rivers in the rainforest were often delayed. Regions like the Central Highlands, which were affected by the high costs of mining production due to transportation difficulties, were a particular priority in the eyes of the government. Primary resources were important to the export economy, but a long list of logistical problems was involved in attempts to move them to the ports (Armas Asín, 2021, p.232-233; Cueto, Contreras, 2008, p.639-644). This reinforced the perception that both the highlands and the rainforest region were the antithesis of the coast. These faraway regions had been populated by “barbaric” races, containing landscapes filled with steep slopes and impenetrable jungles that covered unexploited natural riches. Travelers were at the mercy of environmental hazards, instead of road networks and ports (La Serna, 2015, p.795-798; Poole, 2000, p.196-202). But these negative attributes did not mean that the physical hardships resulting from the geography inhibited anyone from following in Raimondi’s footsteps. On the contrary, Carranza lauded the naturalist’s difficult travels and also viewed the *cordillera* as a place that catalyzed scientific achievements and national identity.

While traveling the route of the unfinished La Oroya railway in 1872, Carranza (1888b, p.46-47) believed the finished railway would open Peru to the world, with “one of the greatest monuments that honored science and human effort.” Completing this project was difficult given the geography, although to Carranza, these “hazards were to be part of the harmonic relationship with Nature, as part of the place where one is born.” The railway was a worthy achievement on the part of a *pueblo* who “knew how to flatten valleys and erect gigantic walls,” but whose human remnants possessed “a profound sadness.” Here Carranza referenced the architectural feats of the Inca civilization, linking the creole technological elite in a genealogical sense with the scientific merits that made the former glory of the Tawantinsuyu possible. He distanced himself and the achievements of men like Raimondi and local communities of knowledge from contemporary native populations who were viewed as homogeneously “sad” (Méndez, 2011, p.82-83). This was in contrast with the other face of such exploration activities, which highlighted the uncomfortable experience of coming into closer contact with the social and political conditions of the inner frontiers of the country.

For Carranza (1887, p.77-79), the second aspect of the heroic narratives of exploration that accompanied Raimondi’s travels also told the sad story of Peru’s internal reality. Considering the many isolated places and densely populated zones in conjunction with inherited “bad habits” and the racial “vices” of the native population, Carranza asserted that while the coastal region was advancing towards progress, the highlands “consumed the social forces [of change] due to the Indigenous people’s intellectual stagnancy and alcoholism.” Resolving this issue would require either leading an awakening in the study of physical phenomena or finding a remedy by “drowning it” (more specifically, the Indigenous population) with European migrants.

It would be decades before ideas regarding race (for example, the notion that certain races were better at intellectual tasks while others excelled at physical work) emerged. Such ideas and the notion of racial mixing to “improve” the offspring of “weak” populations and their productive capacities were not widely discussed in Peru until the turn of the century, but some individuals preceded such debates with similar concerns regarding society and politics. During the development of racial science in the nineteenth century, defining preconceptions that could explain differences in reasoning ability and anthropometric measurements was instrumental in justifying the prevalent social, political, and economic order. Moreover, the old debate on the acclimatization of races around the world defined global populations, in turn nurturing the moral and geopolitical aspects of modern Western thought (Livingstone, 2002, p.159-180; Tucker, 1994, chap.1). Within the postcolonial dilemmas of Republican Peru, the goal of improving the racial pool by encouraging immigration also countered negative impressions that placed Peru in the “torrid zone.” Covering almost a third of the planet’s surface, the Tropics were long believed by Anglo-European scientists as an unsuitable place for white men. Not only did this have practical implications in attracting desired human groups to populate the Andean lands, but it also opened a window for global scientific recognition that the geographical, geological, and meteorological conditions in Peru were unique and that its climes could be a buttress for “progress.”

## Beyond the “pathologized” tropics: in defense of the Peruvian climate

Amid the optimism of the Guano Era, physician, and future *civilista* Francisco Rosas was skeptical that Peru would benefit from assuming a more prominent role in the global networks of commerce. Rosas was aware that such connections increased the chances of bringing in diseases from abroad, and he criticized the lack of attention to hygiene practices and questions of public health caused by the “false belief” that Peru had “one of the healthiest climates in the world.” Improvements in sanitation and public health would be a means to “enhance” the country’s climes, in his opinion (Rosas, 1856, p.11-12). The specter of the insalubrious “torrid zone,” where acclimatization was considered difficult, imposed a medical topography of global standards and external determinants of health that pathologized entire regions and fixed racial distinctions between Europe and the rest of the world (Benson, 2020, chap.2; Harrison, 1999, p.112-123).

In Republican Peru, general concerns about hygiene and the state of public health prevailed because the country lacked a professional body capable of implementing medical policies across the territory. Moreover, there was a strong tendency to rely on premodern methods of administering health care. Some changes were made, as revenues from the guano deposits allowed various advancements in public hygiene for the coastal urban areas. For example, colonial-era hospitals, traditionally run by Public Beneficence Societies, had some architectural enhancements according to novel hygienic principles. Nevertheless, these improvements were mainly limited to Lima, and physicians and caregivers continued to be insufficient within the context of epidemics (Cueto, Palmer, 2014, chap.2; Lossio, 2002, p.82-88).

As the primary export model expanded across Latin America during the second half of the nineteenth century, immigration was believed to be capable of providing the manpower needed for agriculture, for example. Immigrants would arrive with civilized “customs” that could then be incorporated into the national social fabric. For Peru, especially during the reign of the Partido Civil, the consequences of the abolition of slavery and commercial expansion led to greater interest among the bourgeoisie in promoting migration of both “desired populations” from Europe and the controversial but still-necessary Chinese coolies. European immigration proved difficult, however, since many people from this region were biased against migrating to tropical environments. These pathogenic tendencies bred skepticism and posed an obstacle to the elites’ desire for racial improvement; old discussions resumed about race, climate, and health and how local and foreign visions differed (Marcone, 1992, p.64-67; Mücke, 2010, p.73-74; Peard, 1999, chap.3).

Carranza wrote an essay published in *La Gaceta Médica* in 1875 in which he argued that the geological features of the Andes were an expression of the historical expansive forces of the Earth, causing great differences in the landscapes between the sea and the deserts. This generated a great variety of climates in the interior zones, in turn leading to two types of conditioned societies: shires that could facilitate progress and regions that were obstacles to the development of civilization. Nevertheless, this also meant that “climate vulnerability” needed to be overcome, since it was now commonly believed in Europe that white populations would not be able to live beyond certain isothermal lines (Carranza, 1887, p.1-2).

These misconceptions regarding climate and geography were backed by the opinions of scientists like the French anti-acclimatization physician Jean-Christian-Marc Boudin (1806-1867), who was responsible for the negative image attributed to the torrid zone (Carranza, 1887, p.3-4). For this chief medical officer of the French army and follower of Humboldtian physiological principles, organisms were immutable and unable to adapt to different climates, a notion proved by the geographical distribution of disease. Boudin believed that the supposed “cosmopolitanism” of human races was not absolute. With the lack of certainty regarding the predominance of hygienic principles in tropical zones and the ecological stratification of local maladies, Boudin questioned whether effective acclimatization for white populations around the globe could be achieved (Caponi, 2007).

Interestingly, the different ideas that Carranza and Boudin raised reflected two different global and local processes, but each deeply affected the conception of geographical thought. In France, military medical officers re-introduced Hippocratic ideas and used modern methodologies to study physical phenomena, which in turn fueled the debate on acclimatization during the 1850s and 1860s. In Peru, the local scientific community was fascinated with the notion that the *cordillera* was a vast wall preventing intercommunication between regions, thus prompting a progressive reconceptualization of the national space (Caponi, 2007, p.16-18; Orlove, 1993, p.316-317). Although tensions between different degrees of legitimacy in knowledge production (whether local or foreign) were nothing new in Peruvian medical history,^3^ the counterpoint between these two epistemological “moments of transition” was fundamental in shaping local, creole-like imagery of the Tropics and the Andean region with roots in the origins of the “New World.”

The idea of tropical space as a defining trait of the geography of former Spanish America had a long history and continued well into the beginning of the colonial period. In the mid-seventeenth century, the Spanish jurist and chronicler Antonio de León Pinelo determined that Eden had been located in the Americas, noting that the region’s climatic diversity was a sign of “tropicality” which led him to consider this territory as one of the most suitable places for human life. A century later, as part of the Bourbon era of botanical explorations, José Celestino Mutis in New Granada and other enlightened creole scientists recognized the existence of ecological niches in different environmental “microcosms” as resulting from the influence of the Andes over geological and geographical features in the region. Local scientists consequently viewed these ecological niches as natural laboratories where correlations between behavior, race, and climate could be studied and “providential” sources of natural wealth could be found (Cañizares-Esguerra, 2006, chap.6; Scott, 2010, p.83-87). Both these expressions of *criollo* knowledge were part of the genealogies of “tropicality” that arose between the early modern and modern eras. Recognition of the many virtues of climate over a wide expanse constituted a continuous reaffirmation of the embeddedness of local values into global trajectories of defining the geography of the “known” world. This offered complex explanations that granted advantages as well as some biases to the Tropics. There was no single or exclusive rationality marked by the European “pathologization” of nature, which rendered parts of the globe “pestilential rather than paradisical” (Gregory, 2001, p.99-101).

The idea of “tropicality” also played a role in the hierarchization of climate for other zones in the Americas like the Caribbean, but not in a rigid sense. This way of thinking was more open regarding health and ecology, which were viewed as part of an exchange between the sociocultural values of local populations and Euro-American travelers. This meant that meteorological perceptions were sensitive to the levels of local social development, instead of remaining stagnant in a deterministic relationship between diseases and climate (Carey, 2011, p.130-135). As such, Carranza critiqued Boudin’s depiction of the Antilles, more specifically the high mortality reported in Martinique’s white population between 1738 and 1769, which Boudin attributed to the climate. Carranza contended that this was the product of prejudice rather than proper research. Because overall statistical data showed that mortality was high in both Black and white populations in Martinique, Carranza (1887, p.6-10) asserted that Boudin chose “one of the unhealthiest places on Earth” to study the impact of acclimatization. In his opinion, Martinique was insalubrious because of its proximity to the miasmatic environments in the coastal zones of Louisiana.

To Boudin, the climate was known to influence living beings in Humboldtian ecology, and mapping the spatial distribution of diseases remained vital in his medical thinking. As with the distribution of plants in Humboldt’s vertical biogeography, Boudin saw the prevalence of several maladies in the upper and lower segments of the mountain ranges as a direct link between health and the capacity of environmental and physical factors to affect organisms (Caponi, 2007, p.28-30). In contrast, while Carranza also considered altitude a determinant factor in distinguishing between healthy and unhealthy places for white men to acclimatize, he also thought that it was necessary to recognize that even though Peru was geographically located within the Tropics, its overall weather was mild and cold. Citing the impressions of French geologist Louis Agassiz about the comfortable temperatures in the Amazon rainforests, he advanced his argument about the positive influence of the *cordillera* in regulating the atmosphere of the Amazonian jungles, which generally signified its hegemony in regulating the meteorological conditions of the Andean lands. He also presented evidence that heat and humidity did not significantly impact European vitality and acclimatization processes. But Carranza’s (1887, p.18-19, 51-52) ideas also remarked that the neglection of basic hygiene principles increased vulnerability to environmental foci of diseases.

Although the efforts led by the Partido Civil to bring large numbers of white immigrants to Peru were unsuccessful, Carranza’s argument attributing the mortality of European populations in the Tropics to public health issues not only preserved a “positive” legacy related to the construction of creole identities during the colonial period but also served as a major justification for developing the interventionist sanitary policy that characterized Latin American States in the second half of the nineteenth century, a notion that figured in previous imperialist designs of population control in colonial societies that were expected to refashion colonial societies according to European terms of “civilization” (Cueto, Palmer, 2014, chap.2; Harrison, 1999, chap.4; Mücke, 2010, p.74). As a result, when the various initiatives to bring in Caucasian populations with their “superior” customs failed, landlords continued to exert pressure to import workers because of the prevalence of capitalist interests. These landlords resigned to importing more workers but not “citizens,” represented in the East Asian workforce that migrated to Latin American countries like Peru during this era. Carranza believed the future consequences of such migration would include racial degeneracy and a public health debacle.

Carranza wrote an article in 1872 originally intended to express his disagreement with some coastal landlords who were pressing for loans to bring more Chinese migrants to serve as laborers. In the article, Carranza (1888b, p.9-14) claimed that bringing “slaves” would not benefit the country, considering that Chinese migrants represented a real sanitary problem and were obstacles to social and racial improvement. But even though similar views on the Chinese population were held by many hygienists and intellectuals (who believed migrant enclaves were disease foci), Carranza’s ideas went mainly unheard (Palma, Ragas, 2018, p.164-169; Stewart, 1951, chap.6). Despite pleas from some members of the intelligentsia and bourgeoisie to halt Asian migration because Chinese migrants succumbed to vices and represented moral degradation because of the prevailing labor regimes that were believed to be legally extinct in a modernizing Peru, the migration projects enacted by the *civilistas* brought in even more Chinese workers to populate the country. More Asian migrants arrived than the Caucasian immigrants from the cold zones of Europe who were considered more ideal; this led to a steep increase in agricultural revenues benefiting northern landlords who had been agricultural producers until the depletion of guano deposits further aggravated the Peruvian tax crisis of the mid-1870s.

The yearning for a steady stream of European migration continued for many decades until the twentieth century, and the scientific racism behind it found many ways to perpetuate within the public sphere. But only after the ravaging of the country during the War of the Pacific in the early 1880s did scientists and political elites redirect their socio-critical gaze to the sectors of society they believed were the reason for the country’s defeat by Chile. Specifically, instead of looking at themselves, they focused on a group they believed had failed to integrate into the nation since the founding of the Republic: the native population.

## The *indígena* as an ecological condition

Imagining “functional” environments for the modernization of Peru involved a continuous effort to distinguish all the components of local nature that were to become useless since they were not part of the vanguard of changes that were to be made through science. As mentioned earlier, Carranza’s scientific discourse derived from geography, natural history, and medicine served to legitimize his authority in supporting modern Western knowledge as the proper way for society to advance. This knowledge generated different utilitarian means that could become a legible language the state could use to colonize “Nature.”

Postcolonial Peruvian scientists who proclaimed themselves the custodians of the epistemic legacy and tradition of the Incas as creole thinkers set themselves atop a perch from which to judge the “inferiority” of the living remnants of an “ancient and civilized” past. If civilization was possible in Peru (contrary to Orientalist and Eurocentric beliefs), a native force of development should have emerged in a geographical area where it was so difficult to thrive. The ecological relationships between natives and the environment forced them to employ agricultural practices to “tame” the verticality of the Andes. Moreover, as a consequence of living in a place with all the world’s climes, the human diversity of the earliest Indigenous peoples surpassed European classification schemes of civilizational progress (Méndez, 2000, p.29-34; Thurner, 2015, p.42-43).

Although this historicist gaze allowed the creole physician Hipólito Unanue and later the Spanish historian Sebastián Lorente (who taught Carranza in the Colegio de Guadalupe) to introduce colonial and postcolonial Peruvian society into a wider trajectory of global historical theory, it was also used as a rational and common-sense diagnostic for domestic social ills. This viewpoint used environmentally determinist criteria to explain the prevalence of premodern traditions and how the strongly acclimatized bodies of the *indígenas* reflected continuity with the past. The prevalence of premodern traditions and physical acclimatizations typified the ecological relationship the Incas had before the Spanish conquest (Larson, 2004, p.64-65).^4^ This not only constituted an ideological expression of a common view that objectively explained the decadence of contemporary Indigenous populations but also reflected a desire to find specific characteristics in the native population that could define them as future objects of civilization, but not as agents of their own “freedom.”

The conflicting stances of different members of Peruvian society during the War of the Pacific and its aftermath unleashed unresolved tensions that had accumulated for decades. Creole elites used paternalistic and repressive measures to incorporate Indians into the Republic, from abolishing indigenous tribute in 1854 to the start of the war in 1879, which eventually reached a boiling point after the Chilean army advanced into Peruvian territory under the leadership of Patricio Lynch. Motivated by patriotic sentiments of belonging and the possibility to become a part of the national guerrilla movement against foreign invaders, many peasants fought in the central and northern highlands to defend the country. At least, they did until the same general who entrusted them to become his “flag soldiers,” Andrés Avelino Cáceres, unleashed a repressive campaign to consolidate the government presence in zones that were believed to be potential sites of undesired political anarchy after the peace treaty was signed in 1883 (Larson, 2004, p.180-185). This fractured the development of a nationalist sentiment between landlords and the peasantry, as the landlords were caught in the crossfire of supporting local resistance and engaging in collaborationism to protect their economic interests.

Given the threat represented by the effervescent peasant mobilization, landlords became instrumental in eroding the political legitimacy of peasant groupings during the concluding stages of the war. After the process of pacification ended, the inhabitants of the region suffered greatly at the hands of the Chilean Army as well as the guerrillas, leading in turn to the collapse of agricultural, mining, and commercial activities in the region and the death of many livestock. Moreover, a generalized devaluation of the national currency derailed any significant initiative for recovery. The period prior to the war had been characterized by limited modernization in productive industries, especially agriculture in the Mantaro Valley, as the abundance of land and small properties did not promote greater economic competition and consolidation, unlike coastal *haciendas* (Armas Asín, 2021, p.221-223; Mallon, 1983, chap.3; Manrique, 1987, p.176-180).

During this period when the supporters of Miguel Iglesias were trying to rally sufficient resources in northern Peru to face Cáceres and his forces during the Civil War of 1884, the government reinstated the indigenous tribute. This exacerbated resentment among the peasantry that had built up after decades of abuse by landlords and authorities. By the time of the rebellion in 1885 led by Pedro Pablo Atusparia, who had engaged in several skirmishes against the government’s Northern Forces of Pacification, Carranza had written an article about the intellectual deficiencies of the Indians. Mark Thurner (1997, p.425-426) has emphasized how the representation of Indians in Carranza’s essay runs contrary to factual reality. Carranza portrayed Indians as stupid individuals with nonviolent psychological traits, a significant contrast with the reality of the armed insurgency in Ancash and the bloody counterinsurgency campaign. Carranza (1887, p.93-95) began by explaining that the Indians, capable of overcoming great fatigue and carrying heavy loads, were docile beings lacking an internal tendency toward violence. But they also lacked artistic skills, since they were nowhere close to replicating the Incas in construction feats like the palace of Vilcashuaman or other giant monuments that served as the most important testimony to their civilization.

Not only did Carranza argue that the intellect of Indigenous people was “halted” as a consequence of the Spanish conquest, and had not “evolved” since the Inca empire (Thurner, 1997, p.426-427), he also understood the past and present condition of the *indígenas* in a convoluted sense that both legitimized the nationalist identify of the lettered elite and perpetuated a racist image of the country’s native population after Peru’s defeat in the war. As Joseph Dager (2009, p.135-137) has stated, the nationalistic pride in the Incan legacy expressed by creole historians of the nineteenth century made them unable to recognize the value of their object of study. By tracking their genealogies back to the Incan past they identified traits they wished to incorporate into the collective memory and national history, but because it was impossible to completely exclude Andean reality it had to be understood in terms of a glorious past, the contemporary descendants of which were now mere shadows of the race and former civilization that preceded them.

Was it possible for the native population to stop being “themselves” in the mind of scientists and the lettered elite? Although racial classifications throughout the nineteenth century in Latin America were eminently cultural and flexible (therefore creating the possibility that a person could “cease to be” an *indígena*), the prevalent scientific racism at the end of the century crystallized some perceived moral traits and psychological conduct as part of physiological and biophysical processes (Earle, 2007, p.170-173). Nevertheless, even for members of the elite like Manuel Pardo, founder of the Partido Civil, the progressive evolution in political thought led him to see Indigenous people as redeemable if they were westernized, although they would also have to account for the historical problems that characterized their condition. This positioned the elites with the power to shape how social improvements should be made and how energies should be focused among the masses (McEvoy, 1994, p.239-249).

The optimistic vision of the Indians among the *civilistas* was also shared by Carranza, and his desire to incorporate the Indians into the modernizing recovery of his country represented broader strategies to fix past mistakes and to follow the proper steps in guiding the construction of a true nation-state. Two years before he penned the essay he wrote during the Atusparia rebellion, Carranza traveled to the Central Highlands to undertake a socio-political and economic analysis of the conditions among the Indigenous peasants and productive industries in the region after the war against Chile. Throughout the journey Carranza noted his impressions, pointing out how the Indians were a decadent race with stagnated social and economic development. He also noted that the ancestors of these people worked as laborers during the time of the Inca Empire and helped their rulers conquer the geographical hazards of the Andes.

Moreover, even despite their contemporary moral decline, this was a human group that could be awakened from their “lethargy” and be useful to the recovery of the country. For Carranza, the Indigenous people were experts in agricultural practices like using controlled fires to protect their crops from frost and making effective use of available space in vertical landscapes for cultivation. This is similar to the impressions of Archibald Smith decades earlier, in which the physician too saw the Indigenous people as a great labor force for mining activities because they had developed strong physical bodies as a result of their acclimatization to the elevated and oxygen-deprived highlands (Carranza, 1888a, p.75-76; Lossio, 2006, p.841-842). In addition, by contrasting the “natural beauty” of the local geography and the poverty of Ayacucho’s *indígenas*, he recognized the historical roots behind the marginalization of the Indian laborer, as well as the reason behind the erosion of productive possibilities for regional development (after the uneven development of the Coast during the Guano Era). Nevertheless, the stark differences in the landscape and the potential to redeem its population represented hope for future self-sufficiency. Concluding with an optimistic tone, Carranza’s vision for the domestic emergence of an export-geared economy eventually laid the foundations for more prosperous regional imagined communities in the next century (Gamarra, 2014, p.122-124; Gootenberg, 1993, p.196-199).

To accomplish these goals, science and technology were instrumentalized to design policies that could help repair these disparities on regional and national scales. But the scientific core where these ideas would be discussed would not be located in the war-torn provinces, but rather within the arena of the *civilista* politicians and new political actors during the Cacerista government, who looked to regularizing institutional methods in order to revitalize and recover national industries (McEvoy, 2017, p.264-265). Thus, many of the political and economic motivations that led to the incorporation of the Andes and its populations into modernizing efforts were in line with the interests of a still-centralized bureaucratic elite. The *criollo* ruling class saw the problems of the Andes as a synonym for the Indian problem, evidenced in their frequent use of tropes such as “integration” and “progress” to define the role of the *cordillera* in their environmental imaginary (Carey, 2014, p.806-807; Méndez, 2011, p.94; Orlove, 1993, p.320-321). More than the possibility for substantive and widespread change in modern Peru, it paved the way for a flood of scientists into the bureaucratic realms of the government in the coming years.

## Final considerations

As I have tried to show throughout this essay, Carranza’s goal in representing idealistic modernizing images of Andean nature and its populations was not part of an isolated or individual effort on the part of this multi-disciplinary scholar and politician. Rather, the changing political and social circumstances of Republican Peru fueled Carranza’s thinking, sharing many points with the *civilista* elite to which he belonged. This allowed him to link science with the envisioned progress of the country. The use of tropes like *indígena* and *cordillera* reflected a simultaneous interiorization of various concepts into a singular phenomenon. His development of personal scientific thought fashioned various concepts in an interdependent manner where the social ills of the Indigenous peoples from the highlands in the postcolonial Peruvian society further ossified their “natural” place inside a hegemonic order.

Carranza criticized many of the problems that plagued his country’s Republican Era with his particular historical gaze and medical training, as well as his own vision as a *provinciano*. But he was unable to distance himself from widely accepted beliefs around the global hierarchization of races in the nineteenth century. His endorsement of the geological and climatic magnificence of the Andes legitimized the country’s past and “nature” while also advocating for the local production of knowledge and the civilizing potential for Indigenous people; but such beliefs and actions also reinforced the idea that only certain types of people could speak scientifically for the objects of study, while others could not. The modern environmental imaginary of Peru’s Andean lands, as I have shown, figuratively transformed “undomesticated” geographical elements into potentially redeemable objects, laying the foundations for future improvements in society through science.

## NOTES

1 De Rivero y Ustáriz (1798-1857) served as Peru’s first minister of mines and public education in 1825.

2 This alludes to the political platform of the Partido Civil, requiring the productive sector of the country to take an active part in the government.

3 For example, the debate between Peruvian *protomédico* José Manuel Valdés and British physician Archibald Smith in 1835 on the use of calomel to cure dysentery.

4 Both the creole physician and *protomédico* in late-colonial Peru José Hipólito Unanue and the Spanish historian, philosopher, and pedagogue Sebastián Lorente shared a common discourse viewing the influence of “clime” and “soil” as part of the attributes defining Inca civilization, which consequently defined the historical past and future of colonial and postcolonial Peru.
